# Influence of the Sun’s Rays in Consumption

**Published:** 1856-10

**Authors:** 


### *Influence of the Sun's Rays in Consumption.*

In discussing the causes of tuberculosis and tubercular pneumonia, Dr. Coventry has the following judicious remarks:

"There is one subject which requires a more extended notice thanit has usually received from our systematic writers. I refer to the influence of the sun's rays. Every physiologist knows how absolutely necessary they are to the growth of plants, and the etiolating effect their absence or withdrawal has upon the complexion. Is it unreasonable to suppose they may have some influence in causing or preventing tuberculosis! It seems well established, that tubercles may be produced in animals by confining them in close and dark apartments, on a meagre diet. Dr. Hall says, that by this means, he produced fatty degeneracy in animals, which he considers analagous to, if not identical with tuberculosis. In the city where I reside, there was an office connected with a large mercantile establishment, so situated that the sun never shone upon it. It was in the rear of the building, with a single window, and that so surrounded with buildings as to exclude the sun. The occupants of that office died one after another, until the proprietors became alarmed, and had the office removed to another part of the building. One of the occupants I attended, when in the last stage of his disease. He entered the office a strong healthy man with no hereditary tendency to the disease, and temperate and regular in all his habits, but in less than two years he was carried, like his predecessors, to the grave, a victim of consumption. In his case I was never able to discover any cause, unless it was his occupying that fatal office, where he was book-keeper."

The question of the *contagiousness* of consumption is discussed as follows:

"*Contagiousness of Consumption.*—The question of the contagiousness of consumption, or the possibility of its being communicated from one person to another, still divides the profession. It is well known that in the South of Europe, it was formerly considered so contagious that the bed and bedding of persons dying from the disease were burned. Morgagni, in his great work, gives this as a reason why so few cures of the disease are reported, in his post-mortem examinations. Most writers in England and this country, consider the disease as non-contagious. That it is not contagious in the same sense as smallpox, measles, &c., is no doubt true, but that a person occupying the same close apartment, or sleeping in the same bed, breathing an atmosphere filled with the exhalation from ulcerated lungs, may not have his system so poisoned as to produce the disease, may well admit of a doubt. The fact that whole families often die of the disease, is familiar to us all, but this is attributed to their having the same hereditary tendency ; but how does it happen that members of the family who are absent and have no intercourse with the sick, usually escape ; and in the case of husband and wife, where there is no constitutional tendency, the result is often the same. I have seen an account of a man, who said that he had not a near relative in the world, and that he had himself been the unwilling cause of their death ; that he had consumption, communicated it to his wife, and she to others, until the whole family were victims, whilst he himself recovered. The late distinguished Professor Willoughby mentioned to me, that a brother of his caught the disease from attending the sick bed of a beloved daughter. He stated that in his family there was no predisposition, and that his brother was a strong, healthy man, but the daughter had inherited the predisposition from her mother. During the illness of the daughter, her father had the principal care of her, was much over her, and soon fell a victim himself to the disease. It is well known that the glanders in the horse is highly contagious, and that it bears a strong analogy to consumption in man. I would not have it inferred, that I believe there is danger of the disease being communicated where proper precaution is used, but I have seen too many melancholy cases where I believed the disease was communicated to permit me to doubt its possibility."—*Med. & Surgical Reporter.*

